# Natural Killer Cells from Allergic Donors Are Defective in Their Response to CCL18 Chemokine

**DOI:** 10.3390/ijms22083879

**Published:** 2021-04-09

**Authors:** Latiffa Amniai, Coline Ple, Mathieu Barrier, Patricia de Nadai, Philippe Marquillies, Han Vorng, Cécile Chenivesse, Anne Tsicopoulos, Catherine Duez

**Affiliations:** 1 U1019–UMR 9017-CIIL-Center for Infection and Immunity of Lille, Institut Pasteur de Lille, University of Lille, CNRS, Inserm, CHU Lille, F-59000 Lille, France; alatiffa@gmail.com (L.A.); coline.ple@pasteur-lille.fr (C.P.); mathieu.barrier@wanadoo.fr (M.B.); patricia.de-nadai@pasteur-lille.fr (P.d.N.); philippe.marquillies@gmail.com (P.M.); han.vorng@wanadoo.fr (H.V.); cecile.chenivesse@chru-lille.fr (C.C.); anne.tsicopoulos@pasteur-lille.fr (A.T.); 2 CHU Lille, Service de Pneumologie et Immuno-Allergologie, Centre de Compétence pour les Maladies Pulmonaires Rares, F-59000 Lille, France

**Keywords:** natural killer cell, CCL18, migration, cytotoxicity, allergy

## Abstract

Natural killer (NK) cells were originally described as cytolytic effector cells, but since then have been recognized to possess regulatory functions on immune responses. Chemokines locate NK cells throughout the body in homeostatic and pathological conditions. They may also directly stimulate immune cells. CCL18 is a constitutive and inducible chemokine involved in allergic diseases. The aim of this study was to evaluate CCL18’s effect on NK cells from allergic and nonallergic donors in terms of both chemotactic and immune effects. Results showed that CCL18 was able to induce migration of NK cells from nonallergic donors in a G-protein-dependent manner, suggesting the involvement of a classical chemokine receptor from the family of seven-transmembrane domain G-protein-coupled receptors. In contrast, NK cells from allergic patients were unresponsive. Similarly, CCL18 was able to induce NK cell cytotoxicity only in nonallergic subjects. Purified NK cells did not express CCR8, one of the receptors described to be involved in CCL18 functions. Finally, the defect in CCL18 response by NK cells from allergic patients was unrelated to a defect in CCL18 binding to NK cells. Overall, our results suggest that some NK cell functions may be defective in allergic diseases.

## 1. Introduction

Natural killer (NK) cells belong to the family of innate lymphoid cells and are endowed with natural cytotoxicity against infected and cancer cells, as well as immunoregulatory functions [1]. NK cell crosstalk with dendritic cells (DCs), macrophages, neutrophils [2,3] and eosinophils [4,5] may indeed affect the outcome of adaptive immune responses and either exacerbate the immune response or promote immune homeostasis. As such, the role of NK cells in allergic diseases, including atopic dermatitis, rhinitis and allergic asthma, is increasingly studied but is still insufficiently understood [6,7]. For example, in allergic asthma, some modifications in the human NK cell phenotype and functions have been shown either in blood or in peripheral tissues [5,8,9,10,11,12]. For instance, patients with allergic asthma exhibit increased frequencies of IL-4-producing NK cells and decreased frequencies of IFN-γ-producing NK cells in the blood [9]. In animal models, contradictory conclusions on the role of NK cells have been drawn depending on the protocol [13,14,15,16]. Indeed, NK cells were found either to be proinflammatory [13,14], to have no effect [16], or to promote resolution of allergic inflammation [15].

In humans, NK cells are characterized as CD56^+^CD3^−^ cells. They can be broadly subdivided into phenotypically and functionally different populations on the basis of their relative expression of CD56 and CD16 markers [17]. CD56^dim^CD16^+^ NK cells are potent cytolytic effector cells, rapidly secreting proinflammatory cytokines such as interferon (IFN)-γ and cytotoxic mediators (granzymes and perforin) following receptor-mediated activation. By contrast, CD56^bright^CD16^−^ NK cells exhibit reduced lytic capacity, although they can produce IFN-γ when stimulated with cytokines [18]. NK cells constitute 5% to 15% of peripheral blood lymphocytes, but are also located throughout the body in nonlymphoid (lung, gut, skin …) as well as lymphoid organs [19]. NK cell traffic in the circulation and toward tissues and lymphoid organs is regulated by chemokines and chemokine receptors, directing the given NK cell subsets to specific sites [20]. Each CD56^dim^ and CD56^bright^ subset expresses its own pattern of chemokine receptors, while both express the receptors CXCR3 and CXCR4 [21]. Further, chemokines may activate NK cells to exert robust cytolytic activity against tumor cells in cytotoxicity assays performed in vitro and in mouse models [22].

CC chemokine ligand 18 (CCL18) is a CC chemokine constitutively highly expressed in human lung and plasma and upregulated during inflammatory and cancer processes in many organs [23]. It was found to be elevated in bronchoalveolar lavage and serum from allergic asthmatic patients [24] and in many allergic conditions, such as atopic dermatitis and allergic rhinitis [25,26,27]. CCL18 exhibits chemotactic activity toward human naive T cells [28], skin-homing memory T cells [25], B lymphocytes [29], immature DC [30], Th2 cells [24], basophils [24], regulatory T cells [31], and NK cells [32]. The chemotactic response to CCL18 has been shown to be pertussis toxin-sensitive, indicating that its receptor is a member of the G-protein-coupled receptor superfamily. Indeed, CCL18 was reported to be an endogenous agonist of the human CCR8 receptor [33]. However, CCR8 is absent from naive T cells, which have the highest ability to migrate in response to CCL18, suggesting that other receptors may bind CCL18. Two other molecules have been found to be functional receptors of CCL18, but only in cancer: PITPNM3 [34] and G protein-coupled receptor 30 (GPR30) [35]. Finally, glycosaminoglycans (GAGs) were identified to be the major binding molecules for CCL18 on the cellular membrane of peripheral blood leucocytes without mediating chemotactic activity [36]. In addition to its chemotactic activity, CCL18 impacts immunity through direct stimulation of immune cells: it generates tolerogenic DC [37] and regulatory T cells [38] and converts monocytes into M2-like macrophages [39].

To evaluate the effects of CCL18 on NK cells in the context of allergy, we compared the chemotactic and cytotoxic response of NK cells from allergic and nonallergic donors. We show that CCL18 induces migration and promotes cytotoxicity of NK cells from nonallergic donors in a G-protein-dependent manner, whereas NK cells from allergic patients are unresponsive. The results reported here outline a deficiency of NK cells in allergic diseases.

## 2. Results

### 2.1. CCL18 Attracts NK Cells from Nonallergic Donors in a G-Protein-Dependent Manner

**Figure 1:**
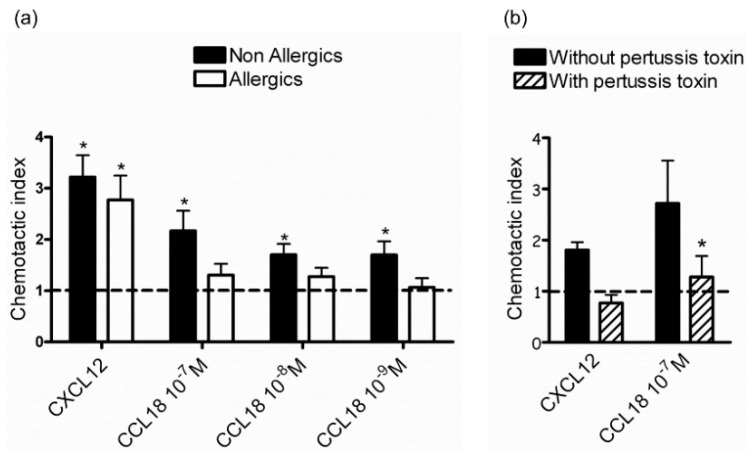
CCL18 attracts Natural Killer (NK) cells from nonallergic donors in a G-protein-dependent manner. (**a**) Migration towards the positive control CXCL12 and different concentrations of CCL18 (*n* = 14 for nonallergic donors, *n* = 5 for allergic donors). The line sets at 1 represent chemotactic index of medium. * *p* < 0.05 chemokines versus medium. (**b**) NK cells from nonallergic donors were incubated with or without pertussis toxin immediately before chemotaxis assay (*n* = 3).

To determine the chemotactic effect of CCL18 on NK cells, in vitro migration was assessed in a Boyden chamber. While NK cells from allergic and nonallergic donors similarly migrated toward the positive control CXCL12 (no significant difference according to two-way ANOVA followed by Sidak’s test), only NK cells from nonallergic donors significantly migrated dose-dependently towards CCL18 (Figure 1a). It has to be noted that despite a significant absence of NK cell migration in allergic patients, no statistical difference was observed between allergic and nonallergic donors. As allergic patients exhibit high levels of circulating CCL18 compared to nonallergic donors [24], which may induce internalization of the receptor after ligand recognition [40], we evaluated NK cell chemotaxis after resting in the absence of ligand. We hypothesized that this would thereby allow receptor cell surface re-expression, as shown for other chemokine receptors [40]. We showed that NK cells from allergic donors cultured in medium alone for 24 and 48 h did not migrate in response to CCL18. We also checked that NK cell migration of nonallergic donors was not due to chemokinesis by performing migration assays in the presence of CCL18 in the upper compartment of the Boyden chamber. No difference in the CXCL12-induced migration was measured between NK cells from allergic and nonallergic donors (Figure 1a), suggesting that CCL18-migration defects on NK cells from allergic donors are restricted to this particular chemokine. Finally, NK cell pretreatment by pertussis toxin abolished the migration of NK cells in response to CCL18 (Figure 1b), suggesting a G-protein-dependent pathway.

**Figure 2:**
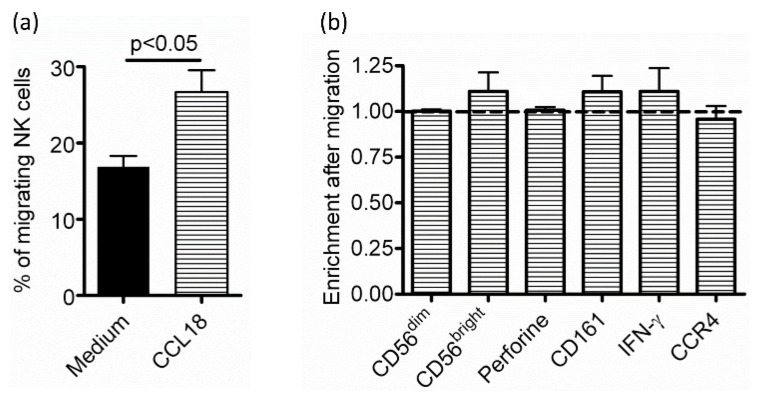
Phenotype of CCL18-recruited NK cells. After overnight culture, the phenotype of NK cells from nonallergic donors (*n* = 6) was analyzed before and after migration towards medium or CCL18 in Transwells. (**a**) Percentage of migrating NK cells. (**b**) With the same donors, enrichment of NK cells in the CD56^dim^CD16^+^, CD56^bright^CD16^−^, perforine^+^, CD161^+^, IFN-γ^+^, CCR4^+^ subsets. Stippled line represents enrichment equal to 1, meaning that the proportion of the NK cell subset was identical before and after migration, which indicates an absence of enrichment.

To evaluate if CCL18 preferentially attracted a defined subset, NK cells from nonallergic donors were stained and analyzed by flow cytometry before and after migration toward CCL18 through a Transwell. As seen in the Boyden chamber, NK cells from nonallergic donors significantly migrated in response to CCL18 (% of migrating cells = 26.69 ± 2.84) compared to migration toward medium (% of migrating cells = 16.88 ± 1.44). However, we did not evidence any enrichment of CD56^bright^ or CD56^dim^ subsets, nor of NK cells positive for perforin or CD161 (involved in cytotoxicity), IFN-γ (the major cytokine produced by NK cells), or CCR4 (important chemokine receptor in Th2 allergic response) (Figure 2). Therefore, we could not define a particular subset of NK cells to be preferentially attracted by CCL18.

### 2.2. CCL18 Induces Cytotoxic Function of NK Cells from Nonallergic Donors in a G-Protein-Dependent Manner But Does Not Stimulate Cytokine Production

**Figure 3:**
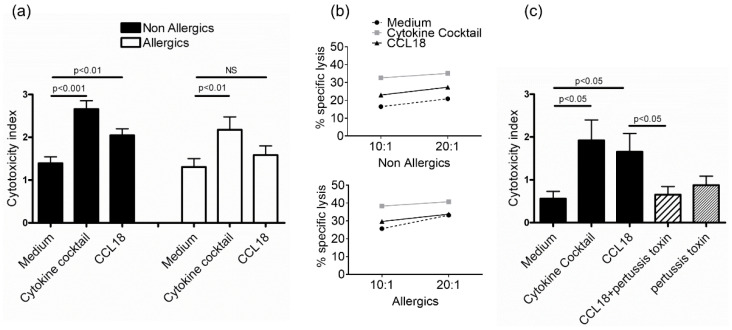
CCL18 enhances cytotoxic activity of NK cells from nonallergic donors in a G-protein-dependent manner. (**a**) Purified NK cells from nonallergic (*n* = 13) or allergic (*n* = 9) donors were incubated overnight in complete medium alone or supplemented with IL-15+IL-12+IL-2 (cytokine cocktail) or CCL18. Afterward, NK cells were incubated with Jurkat target cells at a 10:1 (effector:target) ratio and specific lysis was evaluated by adenylate kinase release. (**b**) NK cells stimulated in the same conditions with cytokine cocktail or CCL18 were incubated with K562 target cells at 10:1 and 20:1 (effector:target) ratio and specific lysis was evaluated by fluorometric method. One representative experiment out of 3 is shown for nonallergic and allergic donors. (**c**) NK cells from nonallergic donors were incubated with or without pertussis toxin immediately before activation with CCL18 (*n* = 3). NS: not significant.

**Figure 4:**
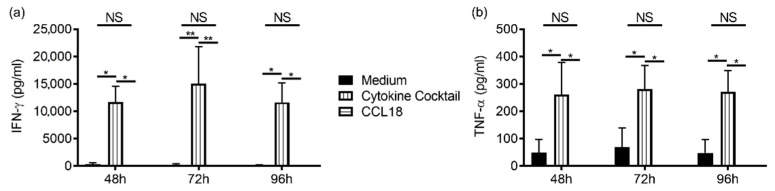
CCL18 does not induce IFN-γ nor TNF-α production by NK cells. Purified NK cells from nonallergic (*n* = 6) donors were cultured in medium or stimulated for 48, 72, and 96 h with cytokine cocktail (IL-2+IL-12+IL-15) or CCL18. IFN-γ (**a**) and TNF-α (**b**) were measured in supernatants. * *p* < 0.05, ** *p* < 0.01, NS: not significant.

Cytotoxicity toward the NK cell-sensitive target cell lines was measured after overnight stimulation of NK cells by CCL18. The cytokine cocktail IL-2+IL-12+IL-15 significantly increased cytotoxicity of NK cells from allergic and nonallergic donors. However, only NK cells from nonallergic donors exhibited increased significant cytotoxicity in response to CCL18 (Figure 3a,b). CCL18-induced cytotoxicity was inhibited by pertussis toxin, suggesting a G-protein-dependent pathway (Figure 3c). In contrast, IFN-γ and TNF-α production was unaffected (Figure 4).

### 2.3. Defective Response of NK Cells from Allergic Patients to CCL18 Is Not Related to Decreased CCL18-Binding

**Figure 5:**
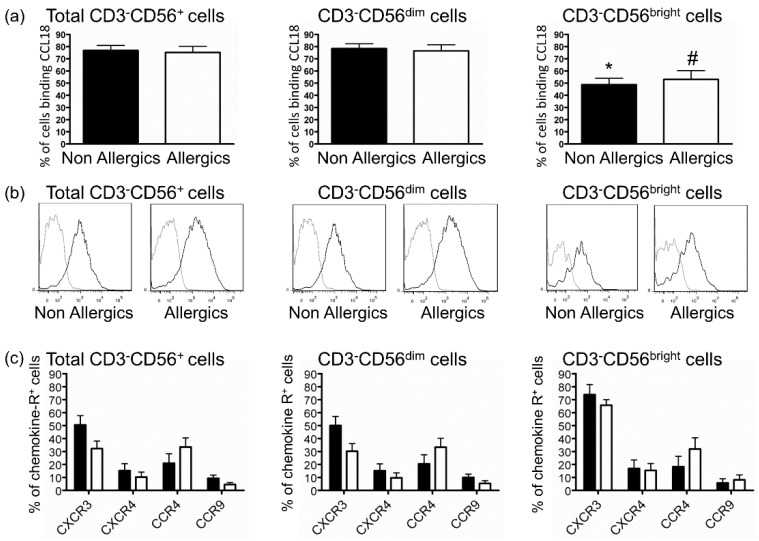
CCL18 binds to NK cells from both allergic and nonallergic donors. (**a**) Mean ± standard error of the mean (SEM) of percentages of cells binding CCL18 among total CD3^−^CD56^+^, CD3^−^CD56^dim^, CD3^−^CD56^bright^ NK cells for nonallergic (*n* = 7) and allergic (*n* = 6) donors. Results are expressed as mean percentage of CCL18 bound positive cells (% of cells binding CCL18) cells ± SEM. * and #: *p* < 0.05 between CD3^−^CD56^dim^ and CD3^−^CD56^bright^ NK cells for nonallergic and allergic donors, respectively. (**b**) A representative histogram of CCL18 binding for each subset is shown for one nonallergic and one allergic donor. Black histogram: biotinylated-CCL18, grey histogram: nonrelevant biotinylated protein. (**c**) Expression of other chemokine receptors on total or subsets of NK cells, isolated from nonallergic (black columns, *n* = 8) or allergic (white columns, *n* = 12) donors. Results are expressed as mean percentage of chemokine receptor positive (% chemokine-R^+^) cells ± SEM.

The defective response of NK cells from allergic donors to CCL18 suggested a problem at the receptor level. Several receptors for CCL18 have been identified, but the only one expressed on peripheral blood leukocytes is CCR8 [32,33]. We were not able to detect CCR8 by flow cytometry on NK cells from either allergic or nonallergic donors, as previously described for unstimulated NK cells [41]. Therefore, we evaluated the capacity of CCL18 to bind to NK cells using biotinylated-CCL18 as previously described [37]. NK cells from both allergic and nonallergic donors were able to highly bind CCL18 (Figure 5a,b). This method measuring CCL18 bound to NK cells certainly revealed high CCL18 binding to GAG as previously shown [32], but was not able to analyze differences in CCL18 G-protein receptor expression. Our results also showed that although both CD56^bright^ and CD56^dim^ NK cell subsets bound CCL18, the number of CD56^dim^ NK cells binding CCL18 was higher than CD56^bright^ NK cell number (Figure 5a,b). Finally, we compared the expressions of other chemokine receptors on NK from allergic and nonallergic donors and found no difference in the expression of CXCR4, corresponding to conserved migration toward CXCL12 (Figure 1a), CXCR3, CCR4 and CCR9 on total NK cells, CD56^bright^ and CD56^dim^ NK cells (Figure 5c).

## 3. Discussion

In this work, we show that CCL18 is able to bind, attract and stimulate NK cells in vitro. This response was restricted to NK cells isolated from nonallergic donors, as NK cells from allergic patients did not respond to CCL18. Such a differential effect has been previously observed for this particular chemokine for T cells and dendritic cells [37]. It might be hypothesized that a desensitization phenomenon of the CCL18 receptor occurs due to the increased level of circulating CCL18 in allergic disease [24]. However, resting of NK cells from allergic patients for 24 or 48 h in medium, which allows re-expression of chemokine receptor after internalization [40], did not restore their in vitro migration, suggesting that CCL18 receptor expression may be downregulated on NK cells in allergic patients. In contrast, CXCL12-mediated migration of NK cells was not affected by allergic status of the donor. Some known chemokine receptors expressed by NK cells, including receptor for CXCL12 (CXCR4), were similarly expressed between allergic and nonallergic donors. These results suggest that the lack of NK cell reactivity to CCL18 in allergy is specific to this chemokine and is not a general migration defect of NK cells. The lack of NK cell reactivity to CCL18 may be related to intrinsic defect in CCL18 receptor expression and function, as previously suggested for T cells [27], or to decreased expression due to chronic allergic inflammation. The low number of patients in each experiment might be a limitation to the study and might explain the absence of statistical difference between allergic and nonallergic donors for response to CCL18, despite the absence of response from NK cells isolated from allergic patients. However, it has to be stated that different donors were used for each experiment (migration in the Boyden chamber and cytotoxicity with adenylate kinase release or fluorimetric method), and each time allergic patients were unresponsive, in contrast to nonallergic donors.

CCL18 putative receptor on NK cells is coupled to G protein as shown by pertussis toxin inhibition of CCL18-induced migration and cytotoxic response. This is concordant with previous studies showing that chemokine signaling in NK cells involves pertussis toxin-sensitive and -insensitive heterotrimeric G-proteins [22,32,42,43]. However, the identification of CCL18 putative receptor on NK cells remains unsolved. Although we show that CCL18 attracts purified NK cells, we were not able to detect CCR8 on purified NK cells, in accordance with a previous study [41]. Labelled CCL18 was able to bind on NK cells isolated from peripheral blood from both allergic and nonallergic donors. However, this result most probably highlights the binding of CCL18 on GAG, molecules covalently linked to a core protein (forming a proteoglycan) on cell surface [32,36], which does not mediate CCL18-induced migration and cytotoxicity of NK cells. This nonetheless suggests that NK cells express proteoglygans not only in their granules [44], but also on their surfaces, and that membrane proteoglygan composition of NK cells might be unaffected in allergy. A decreased proportion of the CD56^bright^ NK cell subset was found to bind CCL18 in comparison to the CD56^dim^ subset, suggesting differential proteoglycan expression between subsets. The role of surface proteoglycan carried by the NK cell membrane is largely unknown, but it has been shown that the intimate interaction of the natural cytotoxic receptor NKp44 and the proteoglycan syndecan-4 in *-cis* can directly regulate membrane distribution of NKp44 and constitutively dampen the triggering of this receptor [45], suggesting a crucial role for membrane proteoglycans in the regulation of NK cell function. In nonallergic donors, CCL18 did not preferentially attract the CD56^bright^ or CD56^dim^ subsets, whereas a larger proportion of the CD56^dim^ subset bound CCL18. This again suggests that binding of CCL18 through GAGs does not account for NK cell migration toward CCL18, and therefore argues for the expression of a putative CCL18 receptor different from GAG. We also showed that CCL18 migration did not modify the percentage of NK cells expressing perforin, CD161, IFN-γ and CCR4. This result suggests that CCL18 does not preferentially attract NK cells specialized in cytotoxicity or IFN-γ production, as shown with the conserved repartition of CD56^bright^ or CD56^dim^ subset after migration, suggesting that both subsets may similarly express the CCL18-receptor. CCL18 also did not preferentially attract NK cells positive for CCR4, an important receptor in Th2 allergic response [46]. 

Finally, CCL18 also increased cytotoxicity of NK cells. Other chemokines have been shown to enhance the cytotoxic activity of resting NK cells mainly through the release of cytotoxic granules [47,48,49]. This granular exocytosis occurred within 4 h. In our study, NK cells were washed after overnight activation with CCL18. Granules may therefore have been washed away and so were not in medium anymore, suggesting that another mechanism may probably account for CCL18-induced NK cell cytotoxicity. One possibility is cell polarization, which is required for the release of NK cell cytolytic granules as well as for the formation of conjugates between killer cells and their target cells, as shown for CCL2, CCL5, CCL3 and CCL4 [50]. Therefore, CCL18 might polarize NK cells, leading to target cell interactions and induction of their lysis. Although usually associated with poor prognosis in cancer, CCL18 expression in tumor sample of gastric cancer was found to predict prolonged survival [51]. Similarly, intratumoral NK cells were associated with prolonged survival of gastric cancer patients [52]. Altogether, this may be related to CCL18 increasing NK cell cytotoxic function.

As CCL18 does not exist in rodents [53], it prevents in vivo studies in animal models. From our results and all previously published articles, we speculate that CCL18 produced in particular by dendritic cells [29,30] may help to locate and activate NK cells in their vicinity for crosstalk [54]. One argument in favor of this hypothesis is given by the analysis of skin lesions of atopic dermatitis patients where NK cells were detected in the epidermis in close contact with CD1a^+^ dendritic cells [54], while all CCL18-expressing cells in the epidermis were CD1a^+^ [25]. Therefore, our results showing that NK cells from allergic donors did not respond to CCL18 may suggest that efficient crosstalk between dendritic cells and NK cells may be disrupted in allergic patients, participating in the defect in the NK cell activation observed in asthma [5,11,12].

## 4. Materials and Methods

### 4.1. NK Cell Preparation

Donors were recruited from the Division of Respiratory, Immunology and Allergy Medicine at the University Hospital of Lille. The project was declared at the Ministère de l’Enseignement supérieur de la Recherche et de l’Innovation under the number DC 2015-2575 and received the approval from the Comité de Protection des Personnes Nord-Ouest (CP 04/45). All patients signed an informed consent form. All allergic patients (gender: 33% females; age: mean 42 years, range 23–53 years) had a clinical history of asthma or rhinitis and exhibited positive skin prick tests toward relevant allergen (80% allergic to house dust mite), positive specific IgE (>3.5 KUI/L). All allergic patients were untreated. Nonallergic patients (gender: 33% females, age: mean 34 years, range 19–50 years) were defined by an absence of allergy history, total IgE levels <100 KUI/L, and negative specific IgE toward major allergens.

Peripheral blood mononuclear cells (PBMCs) were purified on Ficoll-Hypaque gradient (Amersham Biosciences, Uppsala, Sweden). NK cells were isolated using the NK cell isolation kit (Miltenyi Biotec, Bergsch Gladbach, Germany) according to the manufacturer’s recommendations. Purity was assessed by flow cytometry (FACScalibur, BD Biosciences, San Diego, CA, USA) and was greater than 95%. After purification, NK cells were cultured overnight in 24 well plates (Corning Incorporated, Acton, MA, USA) at 10^6^ cells/mL in complete RPMI (RPMI 1640 (Gibco BRL, Thermofisher Scientific, Waltham, MA, USA) supplemented with 10% fetal calf serum, 1% ticarpen, 2 mM L-glutamine) with or without pertussis toxin (0.1 µg/mL, Calbiochem, EMD Chemicals Inc., San Diego, CA, USA).

### 4.2. Staining of NK Cells

10^6^ PBMCs were stained with monoclonal antibodies against BV510-conjugated CD3 (Biolegend, San Diego, CA, USA) and phycoerythrin (PE)-conjugated CD56 (Beckman Coulter, Fullerton, CA, USA). After washing, CCL18 binding on NK cells was performed as previously described [37]. As a control, we used a biotinylated small molecular weight protein derived from Plasmodium falciparum [38]. The specificity of the biotinylated CCL18 binding was checked by inhibition with nonlabeled CCL18. After washings, cells were fixed in paraformaldehyde 1% and analyzed by flow cytometry (LSRFortessa, BD Biosciences, San Jose, CA, USA).

The purity and phenotype of NK cell preparations were measured by cell surface staining with fluorescein (FITC)-, PE- or allophycocyanin-conjugated antibodies against CD56 (Beckman Coulter, Brea, CA, USA), CD3, CD14, CD16, CD19, CD161, IFN-γ (BD Biosciences) and the matched isotype IgG. For the expression of chemokine receptors by NK cells, FITC-conjugated CXCR3, CXCR4, CCR4 and CCR9 (R&D systems, Minneapolis, MN, USA)-specific antibodies were used. After washings, NK cells were immediately analyzed by flow cytometry (FACScalibur, BD Biosciences). Results are expressed as mean percentage of positive cells ± SEM.

### 4.3. NK Cell Migration Assays

Chemotaxis experiments were performed in a Boyden chamber as previously described [31]. NK cells were incubated for 2 h with CCL18 (at a concentration of 10^–10^ to 10^–7^ M, R&D systems), or CXCL12 (10^−7^ M, Peprotech Inc, Rocky Hill, NJ, USA) and complete RPMI as positive and negative controls, respectively. Each condition was performed in triplicate. NK cells were counted in the inferior chamber using a hemocytometer. Results are expressed as chemotactic index = (migration towards the tested chemokine)/(migration obtained with complete RPMI).

To phenotype NK cells migrating in response to CCL18, chemotaxis experiments were performed using Transwells (5 µm, 24 wells, Corning). CCL18 (5 × 10^−8^ M) was added to the lower chamber in a 500 µL volume. A total of 5 × 10^5^ NK cells/100 µL were added to the upper chamber and incubated for 2 h at 37 °C in 5% CO_2_. Migrating NK cells were obtained from the lower chamber and stained for phenotype analysis. NK cell phenotype was also assessed before migration. Results are expressed as percentage of migrating NK cells = (migrating NK cell number)/(NK cell number before migration) × 100, or enrichment after migration = (% of NK cells positive for the marker after migration)/(% of NK cells positive for the marker before migration).

### 4.4. Cytotoxicity Assays

Target cell death was measured either using fluorometric assessment derived from previously described Fluorometric assessment of T lymphocyte antigen specific lysis (FATAL) assay [55] or by measuring the release of the adenylate kinase enzyme from damaged cells (Toxilight™ BioAssay kit, Lonza, Rockland, ME, USA). NK cells were cultured overnight in 6-well plates at 10^6^ cells/mL in complete RPMI (negative control), with cytokine cocktail (IL-15, 20 ng/mL, plus IL-12, 5 ng/mL, plus IL-2, 10 ng/mL) (positive control), or with CCL18 (10^−8^ M). Target cell lines (K562 and Jurkat cells) were from the American Type Culture Collection (Manassas, VA, USA).

For FATAL assay, K562 cells were stained with 2 × 10^−6^ M PKH-26 (Sigma-Aldrich, Saint-Louis, MO, USA) and 2.5 × 10^−6^ M carboxyfluorescein diacetatesuccinimidyl ester (CFSE) (FluoProbes, Scottsdale, AZ, USA). Target cells and NK cells were incubated for 3 h at different effector:target (0:1, 10:1, 20:1) ratios in a 96-well round bottom plate (Corning, Corning, NY, USA). The death of PKH-26^+^ target cells was measured by intracellular CFSE loss on flow cytometer (FACScalibur, BD Biosciences). Cytotoxicity is expressed as % specific lysis = (% CFSE^low^ cells obtained in the tested ratio)−(% CFSE^low^ cells obtained for target cells alone (ratio 0:1)).

For measurement of adenylate kinase release, Jurkat (sensitive to Fas-dependent cytotoxicity) and NK cells were resuspended at 0.5 × 10^6^ and 10 × 10^6^ cells/mL, respectively, and incubated for 3 h at 37 °C in 5% CO_2_ at a 10:1 effector:target ratio in a 96-well round bottom plate. To monitor NK cell death, NK cells were incubated alone in complete RPMI in numbers equivalent to a ratio 10:1. Cell death was evaluated by the Toxilight BioAssay on supernatants according to the manufacturer’s recommendations and measured as count per minute (cpm) (Victor, PerkinElmer, Waltham, MA, USA). Results are expressed as cytotoxicity index = (cpm obtained for the tested ratio in wells containing target and NK cells−cpm obtained for the corresponding ratio in wells containing NK cells alone)/mean cpm for target cells alone.

### 4.5. IFN-γ and TNF-α Production Measurement

Purified NK cells (10^6^/mL) were cultured in a 96-well plate (BD biosciences) in complete RPMI with cytokine cocktail, composed of IL-2 (10 ng/ml) + IL-12 (5 ng/mL) + IL-15 (10 ng/mL), or CCL18 (10^−8^ M) for 48, 72 and 96 h. Supernatants were collected and levels of IFN-γ and TNF-α were measured by ELISA according to manufacturer recommendations (Diaclone, Besançon, France). Results are expressed as pg/mL.

### 4.6. Statistical Analysis

Values for all measurements were expressed as the mean ± standard error of the mean (SEM). Statistical analyses were carried out using GraphPad Prism 4.03 software (Graphpad, San Diego, CA, USA). Normality tests were performed and were either followed by Wilcoxon, one-way or two-way ANOVA tests, and post-tests when necessary (Sidak, Tukey, Friedman). Significance for *p* values was set to 0.05.

## Data Availability

Data are available upon reasonable request from the corresponding author.
